# Design and characterisation of a film-forming emulsion combining enhanced skin permeability with prolonged drug delivery

**DOI:** 10.1016/j.bbiosy.2026.100144

**Published:** 2026-08-03

**Authors:** Jia Hu, Jannatul Fardous, Yi Zhang, Yasuhiro Ikegami, Hiroyuki Ijima

**Affiliations:** a Department of Chemical Engineering, Faculty of Engineering, Graduate School, Kyushu University, 744 Motooka, Nishi-ku, Fukuoka, 819-0395, Japan; b Department of Pharmacy, Faculty of Science, Comilla University, Comilla, 3506, Bangladesh

**Keywords:** Film-forming emulsion, Water-in-oil nanoemulsion, Transdermal drug delivery, Permeability

## Abstract

Water-in-oil nanoemulsions can improve the skin permeation of encapsulated hydrophilic drugs; however, their limited residence on the skin restricts prolonged drug delivery. To address this limitation, we developed a novel film-forming emulsion (FFE) by incorporating our previously developed water-in-oil nanoemulsion (W_G_/O-NE) into an *in situ* film-forming matrix, thereby combining the penetration-enhancing function of W_G_/O-NE with prolonged skin residence. W_G_/O-NE with a mean particle size of 141.2 ± 0.6 nm was incorporated into the matrix to produce FFE droplets with a mean diameter of 1.02 ± 0.47 μm. Formulation optimisation identified 10% (w/v) PVA and 10% (v/v) glycerol as balancing skin permeability and adhesion. FFE formed a flexible, adherent film. Confocal laser scanning and scanning electron microscopy revealed interconnected porous structures, and W_G_/O-NE released from the dried film retained a nanoscale particle size distribution. In an *in vivo* mouse skin penetration study, FFE increased skin retention of fluorescein, a hydrophilic model drug, by 2.31- and 3.99-fold compared with the film-forming system and W_G_/O-NE, respectively, and enabled delivery to subcutaneous tissue at depths of ≥375 μm. Fluorescein remained detectable for up to 48 h under repeated application. Safety evaluation revealed no obvious tissue damage, and transient epidermal thickening resolved within 2 days after film removal. These findings suggest that W_G_/O-NE can be released from the FFE film and that FFE may combine film-mediated skin retention with the release and penetration-enhancing functions of W_G_/O-NE. Thus, FFE may serve as a platform for prolonged skin residence and deep delivery of hydrophilic drugs.

## Highlights

• Intact W_G_/O-NE was encapsulated within a hydrophilic film-forming matrix.
• FFE formed internal emulsion pathways that promoted drug release and transport.
• FFE doubled skin retention *vs* FFS and quadrupled it *vs* W_G_/O-NE in mice.
• FFE delivered a hydrophilic model drug into subcutaneous tissue (>375 µm).

## Graphical abstract

**Figure:**
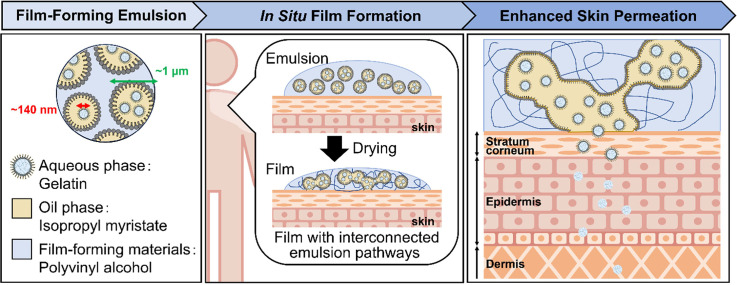

## 1 Introduction

Transdermal drug delivery systems (TDDS) have attracted significant attention owing to their non-invasive nature, avoidance of first-pass metabolism, and reduced systemic side effects [1,2]. However, the stratum corneum acts as a strong hydrophobic barrier that severely limits the transdermal delivery of hydrophilic and high-molecular-weight drugs (>500 Da) [3]. Various carriers, such as liposomes, microneedles, and nanocarriers, have been investigated to overcome this barrier. Water-in-oil nanoemulsions (W/O-NEs) have been developed to improve the skin permeation of hydrophilic drugs, as the physicochemical properties of the continuous oil phase play a critical role in transdermal transport. Isopropyl myristate (IPM), a commonly employed oil phase and well-established penetration enhancer [4], has been reported to exhibit high affinity for the intercellular lipid matrix of the stratum corneum. Through favourable lipid–lipid interactions, IPM may partition into and perturb lipid lamellae, increasing lipid fluidity and thereby facilitating drug diffusion via the intercellular pathway [5,6]. In a previous study, a novel Water-in-oil nanoemulsion (W_G_/O-NE) was developed using IPM as the continuous oil phase. W_G_/O-NE exhibited favourable physicochemical properties, including a suitable particle size of approximately 100 nm and excellent storage stability [7]. In addition, optimised W_G_/O-NE significantly enhanced transdermal penetration into the epidermis and enabled intradermal delivery of growth factors [8,9].

However, most nanoemulsion-based transdermal formulations have been designed primarily to enhance skin permeation, whereas their ability to provide prolonged skin residence and controlled release remains limited [10]. To overcome this limitation, nanoemulsions have been incorporated into semi-solid or film-forming dosage forms, such as nanoemulgels and film-forming systems (FFS), to increase formulation viscosity, improve residence time on the skin, and sustain drug release [11,12]. In particular, film-forming emulsions (FFE) have recently attracted attention as *in situ* film-forming formulations, in which emulsion droplets are dispersed within a polymeric matrix after solvent evaporation [13]. Previous FFE studies have mainly focused on oil-in-water (O/W) emulsions, where oil droplets containing lipophilic drugs are incorporated into a dry film-forming polymer matrix [14,15]. In contrast, the incorporation of W/O-type nanoemulsions, especially W_G_/O-NE with an oil-continuous external phase, into a hydrophilic film-forming matrix remains challenging because of the incompatibility between the oil-continuous nanoemulsion and the aqueous polymer phase. Moreover, although previous studies have evaluated the skin permeation performance of nanoemulsion-based films, limited attention has been paid to the internal film structure, the structural integrity of incorporated nanoemulsions after film formation, and their release behavior from the film matrix.

Therefore, this study aimed to develop a transdermal formulation that integrates the penetration-enhancing capability of W_G_/O-NE with prolonged skin residence provided by an *in situ* film-forming matrix. To this end, we designed a Water-in-oil-in-water (W/O/W)-type FFE by incorporating W_G_/O-NE into a hydrophilic polymer matrix, as shown in Fig. S1. We hypothesised that the dispersed oil-continuous W_G_/O-NE phase could contribute to the formation of an internal “emulsion pathway” within the dried film matrix, which may facilitate drug release while maintaining the permeation-enhancing function of W_G_/O-NE. Accordingly, the present study evaluated the formulation characteristics, internal film structure, release behaviour, skin penetration performance, and biocompatibility of the FFE system.

## 2 Materials and methods

### 2.1 Materials

Porcine-skin-derived gelatin (Type A) was purchased from Sigma–Aldrich (St. Louis, MO, USA). IPM, PVA (degree of polymerisation 1500–1800), glycerol, and N-hydroxysuccinimide (NHS) were purchased from Fujifilm Wako Pure Chemical Industries Ltd. (Osaka, Japan). 1-Ethyl-3-(3-dimethylaminopropyl)carbodiimide hydrochloride (EDC) was purchased from Peptide Institute, Inc. (Osaka, Japan). 2-Morpholinoethanesulfonic acid monohydrate (MES) was purchased from Dojindo Laboratories (Kumamoto, Japan). Sucrose erucic acid ester (ER-290) was provided by Mitsubishi Chemical Foods Co. (Tokyo, Japan). Polyoxyethylene hydrogenated castor oil (HCO-60) was kindly provided by Nihon Surfactant Kogyo Co., Ltd. (Otawara, Tochigi, Japan). Absolute ethanol (Traceable 99, 1st grade) was purchased from Nihon Alcohol Hanbai Co., Ltd. (Tokyo, Japan). Coumarin 6 (C6) was purchased from Tokyo Chemical Industry Co., Ltd. (Tokyo, Japan), and fluorescein sodium (FL; FLUORESCITE®) was obtained from Alcon Pharma (Tokyo, Japan).

### 2.2 Animals

ICR mice (Institute of Cancer Research mice, male, 6 weeks old, Japan SLC, Shizuoka, Japan) were housed in cages and had access to a standard diet *ad libitum*. The experimental procedures followed the protocols approved by the Ethics Committee on Animal Experiments of Kyushu University (A9-381-0).

### 2.3 Preparation of W_G_/O-NE

W_G_/O-NE was prepared based on previous studies [7,8]. ER-290 50 mg/mL was added to IPM to prepare the oil phase. The EDC/NHS solution was prepared by adding 10 mg/mL EDC and 6 mg/mL NHS to 0.05 M MES buffer. The EDC/NHS solution was added to 5% (w/v) gelatin solution to prepare the aqueous phase. The aqueous phase was mixed with the oil phase and emulsified using an ultrasonic homogeniser (Sonifier 250, Branson Ultrasonics Co., Danbury, CT, USA) at 70% amplitude with pulsed sonication (1 pulse/0.7 s) in an ice-water bath to minimise temperature rise during emulsification. The resulting emulsion was subsequently cooled at 4 °C to gel the inner aqueous phase, yielding W_G_/O-NE. The formulation composition is summarised in Table S1.

### 2.4 Preparation of FFE

The multiple W/O/W emulsion was prepared by vortexing W_G_/O-NE, 50 mg/mL HCO-60 solution, and absolute ethanol in a volumetric ratio of 1:1:2 for 15 s. The resulting mixture was immediately added to a PVA solution under continuous stirring, where the PVA solution volume was set to 1.5-fold the total volume of the initial mixture, followed by the addition of glycerol. The solution was continuously stirred for more than 10 min to obtain FFE. To prepare the FFE film, the obtained FFE was evenly spread as a thin layer on a glass plate or skin surface and dried for at least 10 min. The formulation composition is summarised in Tables S2 and S3.

### 2.5 Characterisation of W_G_/O-NE

#### 2.5.1 Particle size measurement of W_G_/O-NE

The mean particle diameter and distribution of W_G_/O-NE were measured using dynamic light scattering (DLS; nanoSAQLA, Otsuka Electronics Co., Ltd., Hirakata, Osaka, Japan). The measurement was performed at 20 °C and a scattering angle of 173° W_G_/O-NE was diluted 600 times with IPM before measuring the particle size.

#### 2.5.2 Encapsulation efficiency (EE) of FL-encapsulated W_G_/O-NE

The EE of FL in W_G_/O-NE was determined by quantifying the amount of non-encapsulated FL separated from the formulation after centrifugation [16]. Briefly, FL-encapsulated W_G_/O-NE was centrifuged at 1780 × g for 10 min, and the upper oil phase was collected. The collected upper phase was mixed with ethanol at a volume ratio of 1:1, and the fluorescence intensity was measured using a microplate reader (SpectraMax Gemini EM, Molecular Devices, San Jose, CA, USA) at excitation and emission wavelengths of 494 and 521 nm, respectively.

A matrix-matched calibration curve of fluorescein sodium was prepared over the concentration range of 7.8–62.5 μg/mL using ethanol/IPM containing 50 mg/mL ER-290 (1:1, v/v), which was the same solvent composition used for sample measurement. Good linearity was obtained with the regression equation y = 324.81x+769.49 (R^2^=0.9981). The concentration of free fluorescein in the upper phase was calculated from the calibration curve.

The EE% was calculated using Eq. (1):

(1) $$\text{EE}\%=\frac{\left(Total\;drug\;added-Non\;encapsulated\;drug\right)}{Total\;drug\;added}\times \;100\%$$

### 2.6 Characterisation of FFE

#### 2.6.1 Droplet size measurement of FFE

To measure the droplet size of the FFE, the oil phase of W_G_/O-NE was labelled with C6, a green hydrophobic fluorescent dye. The prepared C6-encapsulated FFE was diluted tenfold with ultrapure water and observed using a CLSM (Leica TCS SP8, Wetzlar, Germany). The droplet size was analysed using ImageJ software.

#### 2.6.2 Morphological characterisation of FFE

The morphological characteristics of the FFE film were examined using a SEM (JCM-7000 NeoScope, Tokyo, Japan) to analyse its surface structure. Additionally, to investigate the three-dimensional distribution of W_G_/O-NE within the FFE, a C6-encapsulated FFE was prepared and its internal structure was visualised using a CLSM.

#### 2.6.3 Measurement of skin adhesion strength of FFE

For the skin adhesion test, porcine ears (FUKUOKA MEAT SALES Co., Ltd. Fukuoka, Japan) containing cartilage were cut into rectangular samples (5 cm × 1.5 cm). The FFE prepared in Section 2.4 was applied onto a 3.5 cm × 1 cm area of the porcine ear skin and dried for 30 min. A portion of the film was peeled off, leaving an exposed adhesive area of 1 cm × 1 cm. The upper side of the film and lower porcine skin were clamped onto a tensile testing machine with a test force range of 10 ×, test speed of 100 mm/min, and sensitivity of 9.9%. The maximum stress required to detach the film from the skin was recorded.

#### 2.6.4 Physical stability of FFE during storage

The physical stability of FFE was evaluated by visual observation of macroscopic phase separation during storage [13]. Freshly prepared FFE was transferred into sealed microcentrifuge tubes and stored undisturbed at 4 °C and 25 °C. At predetermined time points (0.5, 1, 2, 4, 24, 48, 72, and 144 h), the samples were photographed and visually inspected for phase separation, creaming, sedimentation, precipitation, or obvious changes in appearance. FFE formulations containing different PVA concentrations were evaluated under the same storage conditions and at the same predetermined time points. Formulations containing different glycerol concentrations were visually evaluated after 72 and 144 h of storage at 4 °C and 25 °C.

#### 2.6.5 Particle size measurement of W_G_/O-NE released from FFE

To evaluate whether W_G_/O-NE retained its nanoscale structure after incorporation into and release from FFE, the particle size distribution of W_G_/O-NE released from the FFE film was measured by DLS [17]. Briefly, freshly prepared FFE was spread on a glass plate and dried for 10 min to form a film. The obtained FFE film was immersed in IPM and incubated under gentle shaking for 24 h. The particle size distribution of the released W_G_/O-NE was measured using the same DLS conditions as described in Section 2.5.1. The particle size distribution of freshly prepared W_G_/O-NE was measured as a control.

### 2.7 *In vitro* FL release from W_G_/O-NE and FFE

*In vitro* release of FL from W_G_/O-NE and FFE formulations was evaluated using a dialysis method [18]. Briefly, samples were placed in dialysis tubing (MWCO: 3.5 kDa) and immersed in PBS under gentle stirring at 37 °C. At predetermined time points (0.5, 1, 2, 4, 8, 24, and 48 h), 1 mL of release medium was collected and replaced with 1 mL of fresh PBS. The concentration of released FL was determined by fluorescence measurement at excitation and emission wavelengths of 494 and 521 nm, respectively, and cumulative release (%) was calculated.

### 2.8 *In vitro* penetration study

Using the green hydrophilic fluorescent dye as a model drug, the *in vitro* penetration of FFE was studied. FL-encapsulated FFE was prepared using the method shown in Section 2.4. The skin was excised from the porcine ears using a scalpel and cut into approximately 2 cm^2^ squares. FL-encapsulated FFE 50 µL was applied onto the skin surface and formed a film. The skin was then placed into diffusion cells (diffusion area of 0.64 cm^2^) and the experiment was conducted at 32 °C for 24 h. After incubation, residual formulation was gently removed by washing the skin surface with PBS. The treated skin was then collected, weighed, minced, and sonicated in PBS to extract FL. Fluorescence intensity was measured using a microplate reader at excitation and emission wavelengths of 494 and 521 nm, respectively. The FL concentration in the extract was determined using a calibration curve. The FL content retained in the skin was normalised to skin weight and expressed as μg/g skin.

### 2.9 *In vivo* penetration study

Mice were acclimated for approximately one week through standard feeding. Subsequently, the dorsal hair of the mice was gently shaved, and 300 µL FL-encapsulated FFE was applied to a 2 cm × 3 cm area on the back. At predetermined time points after application (1, 6, 24, and 48 h), the mice were euthanized. The residual formulation on the skin surface was gently removed by washing the skin surface with PBS, and the treated skin area was collected. For the 1, 6, and 24 h groups, skin samples were collected after a single FFE application. For the 48 h group, the FFE film was replaced once at 24 h, and the skin was collected 24 h after the second application. The skin samples were embedded in optimal cutting temperature (O.C.T.) compound (Sakura Finetek USA, Inc., Torrance, CA, USA) and frozen at −80 °C until cryosectioning. The frozen skin was cut to a thickness of 20 µm using a microtome (LEICA CM1860 UV, Leica Microsystems K.K, Tokyo, Japan) and placed on a glass slide, followed by observation using a CLSM. For comparison, FL-encapsulated W_G_/O-NE and an FL-containing FFS were prepared and evaluated under identical experimental conditions. The FFS was prepared using the same final PVA concentration of 10% (w/v) and glycerol concentration of 10% (v/v) as the optimised FFE formulation, with FL directly dissolved in the film-forming matrix. All three formulations (W_G_/O-NE, FFS, and FFE) contained FL at the same final concentration. The fluorescence penetration distance was quantitatively analysed using ImageJ software. Regions containing hair follicles were excluded from the analysis to minimise the contribution of follicular penetration pathways. For quantitative analysis of FL retained in the skin, the recovered treated skin samples were weighed, minced, and sonicated in PBS to extract FL, using the same extraction procedure as described in Section 2.8. The FL concentration in the extract was determined using a calibration curve, normalised to skin weight, and expressed as μg/g skin.

### 2.10 Biocompatibility

Skin samples from the experiment described in Section 2.9 were collected and processed for histological analysis. The excised skin was fixed in 10% neutral-buffered formalin for 24 h, dehydrated using a graded ethanol series and embedded in paraffin. Paraffin-embedded sections were stained with haematoxylin and eosin (H&E) and examined under a light microscope (CKX53; Olympus, Tokyo, Japan) to assess tissue morphology. To quantitatively evaluate potential changes in the skin structure, the thicknesses of the epidermal and dermal layers were measured using ImageJ software based on histological images.

To evaluate the skin recovery ability after FFE administration, it was applied to the skin of mice for 24 h. After application, the film was carefully peeled off and the animals were maintained under normal housing conditions for an additional 2 d without further treatment. Subsequently, the treated skin areas were excised and subjected to H&E staining to observe histological changes. Epidermal thickness was quantitatively analysed using ImageJ software. In addition, skin moisture content was measured daily, and skin hydration capacity was assessed using a skin evaporative water logger (SR-101, LOZENSTAR Co., Ltd., Osaka, Japan).

### 2.11 Statistical analysis

Data are presented as the mean ± standard deviation (SD). Differences among multiple groups were analysed using one-way analysis of variance (ANOVA), followed by Tukey’s HSD post hoc test. Statistical analyses were performed using an online statistical calculator [19]. A *p*-value of less than 0.05 was considered statistically significant.

## 3 Results

### 3.1 Preparation and characterisation of W_G_/O-NE and FFE

**Fig. 1:**
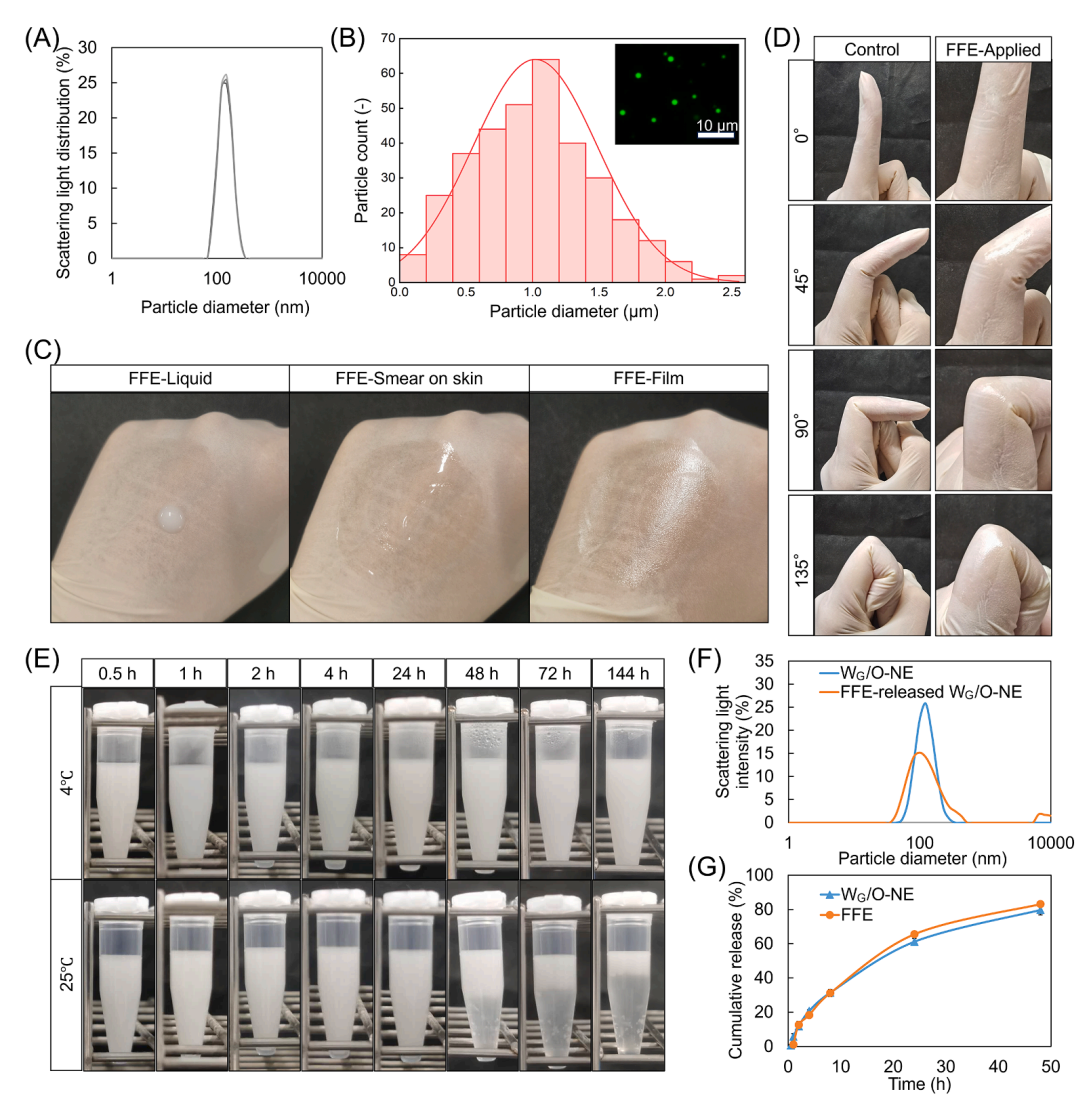
Characterisation of W_G_/O-NE and FFE. (A) Particle size distribution of W_G_/O-NE, *n* = 3. (B) Droplet size distribution of FFE, Bar: 10 µm. (C) Application of FFE. (D) Flexibility and adhesion of the FFE film at different finger bending angles. (E) Representative photographs of FFE stored at 4 °C and 25 °C for up to 144 h for visual assessment of short-term macroscopic physical stability. (F) Particle size distributions of W_G_/O-NE before incorporation into FFE and W_G_/O-NE released from FFE. (G) Cumulative release profiles of fluorescein sodium from W_G_/O-NE and FFE in PBS, *n* = 3. W_G_/O-NE: water-in-oil nanoemulsion; FFE: film-forming emulsion.

The particle size distribution of W_G_/O-NE is shown in Fig. 1A. The average particle size was determined to be 141.2 ± 0.6 nm, with a polydispersity index (PDI) of 0.013 ± 0.022. The droplet size distributions of the FFE are shown in Fig. 1B The average droplet size was determined to be 1.02 ± 0.47 µm. The EE of FL in W_G_/O-NE, calculated using Eq. (1), was 94.6 ± 0.4%.

Fig. 1C illustrates the appearance of the FFE applied to the back of the hand (with gloves). Within 10 min of application, the FFE formed a transparent film on the glove surface, closely conforming to the contours of the skin, demonstrating excellent film-forming ability and skin adhesion. As shown in Fig. 1D, the FFE film exhibited excellent flexibility and adhesion when applied to the finger joints. The film maintained its integrity and conformed well to skin movement under different bending angles (0°, 45°, 90°, and 135°) without cracking or peeling.

The physical stability of FFE was evaluated at 4 °C and 25 °C for up to 144 h. As shown in Fig. 1E, no obvious macroscopic phase separation, creaming, sedimentation, or precipitation was observed during storage at 4 °C. In contrast, creaming was observed in the samples stored at 25 °C after 48 h. An ethanol-challenge experiment using a simplified PVA-free W/O/W nanoemulsion model prepared with the same W_G_/O-NE as FFE showed no marked destabilisation at ethanol concentrations of up to 60% (v/v) over 10 days (Fig. S2). The particle size distribution of W_G_/O-NE released from the FFE film was then compared with that of freshly prepared W_G_/O-NE. As shown in Fig. 1F, the released W_G_/O-NE showed a nanoscale particle size distribution similar to that of freshly prepared W_G_/O-NE, although the peak intensity was slightly lower and broader after release from the FFE film. The *in vitro* FL release profiles of W_G_/O-NE and FFE are shown in Fig. 1G Both formulations showed time-dependent FL release in PBS at 37 °C, with cumulative release reaching approximately 60–65% at 24 h and approximately 80% at 48 h. The release profile of FFE was comparable to that of W_G_/O-NE.

### 3.2 Optimisation of PVA concentration in FFE

**Fig. 2:**
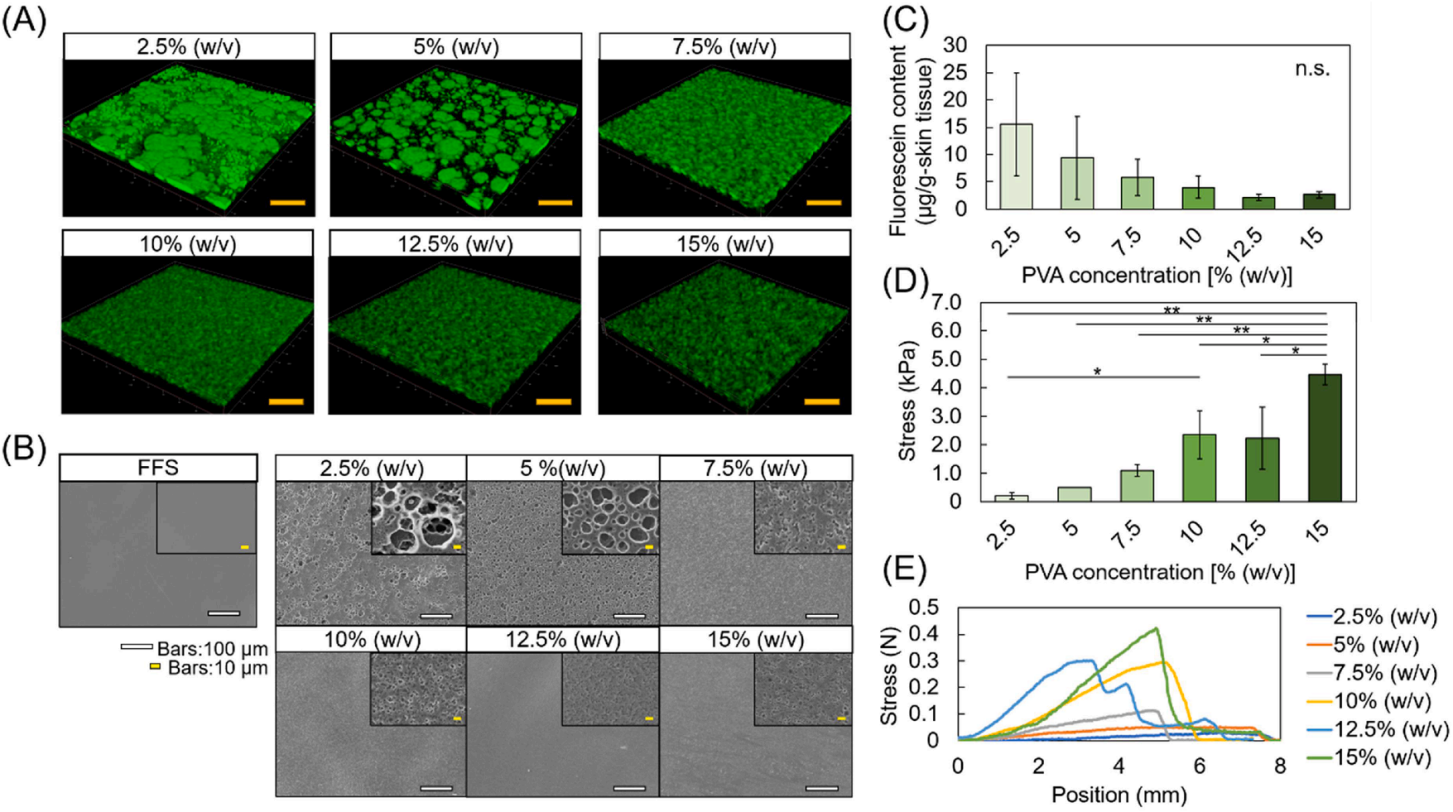
Optimisation of PVA concentration in FFE. (A) CLSM images of FFE film, Bars: 50 µm. (B) SEM images of FFS/FFE film on the skin contact surface, Bars: 100 µm (white), Bars: 10 µm (yellow). (C) Skin penetration of encapsulated FL from FFE at different PVA concentrations, *n* = 3, n.s. = not significant. (D) Skin adhesion of FFE at different PVA concentrations, *n* = 2, *p < 0.05; **p < 0.01. (E) Stress-strain curve of FFE at different PVA concentrations. PVA: polyvinyl alcohol; CLSM: confocal laser scanning microscopy; SEM: scanning electron microscopy; FFE: film-forming emulsion; FFS: film-forming system; FL: fluorescein sodium.

We subsequently investigated the effects of PVA concentration on the stability, structure, skin adhesion, and *in vitro* skin penetration of encapsulated FL from FFE. Specifically, PVA concentrations of 2.5, 5.0, 7.5, 10, 12.5, and 15% w/v were tested. The short-term physical stability of FFE was visually evaluated during storage. Macroscopic creaming was less evident in formulations with higher PVA concentrations and in samples stored at 4 °C, whereas lower PVA concentrations showed more obvious creaming at 25 °C (Fig. S3). The results of CLSM analysis are shown in Fig. 2A. The green fluorescence indicates the distribution of W_G_/O-NE droplets within the FFE film. Significant aggregation of the W_G_/O-NE droplets was observed in samples containing 2.5% and 5% PVA. In contrast, the samples with PVA concentrations of 7.5% or higher exhibited a more uniform distribution of smaller W_G_/O-NE droplets within the FFE film, forming interconnected pathway structures.

The SEM images are shown in Fig. 2B No visible pores are detected in the FFS group, indicating a dense microstructure. In contrast, all the samples in the FFE group exhibited interconnected porous networks. Larger pores were observed in the 2.5% and 5% samples, whereas smaller pores appeared at PVA concentrations of 7.5% and above.

As shown in Fig. 2C, skin penetration of the encapsulated model drug tended to decrease with increasing PVA concentration; however, the difference was not statistically significant. The adhesion data in Fig. 2D and the stress–position curves in Fig. 2E show that the maximum stress required to peel off the film increased with increasing PVA concentration.

### 3.3 Optimisation of glycerol concentration in FFE

**Fig. 3:**
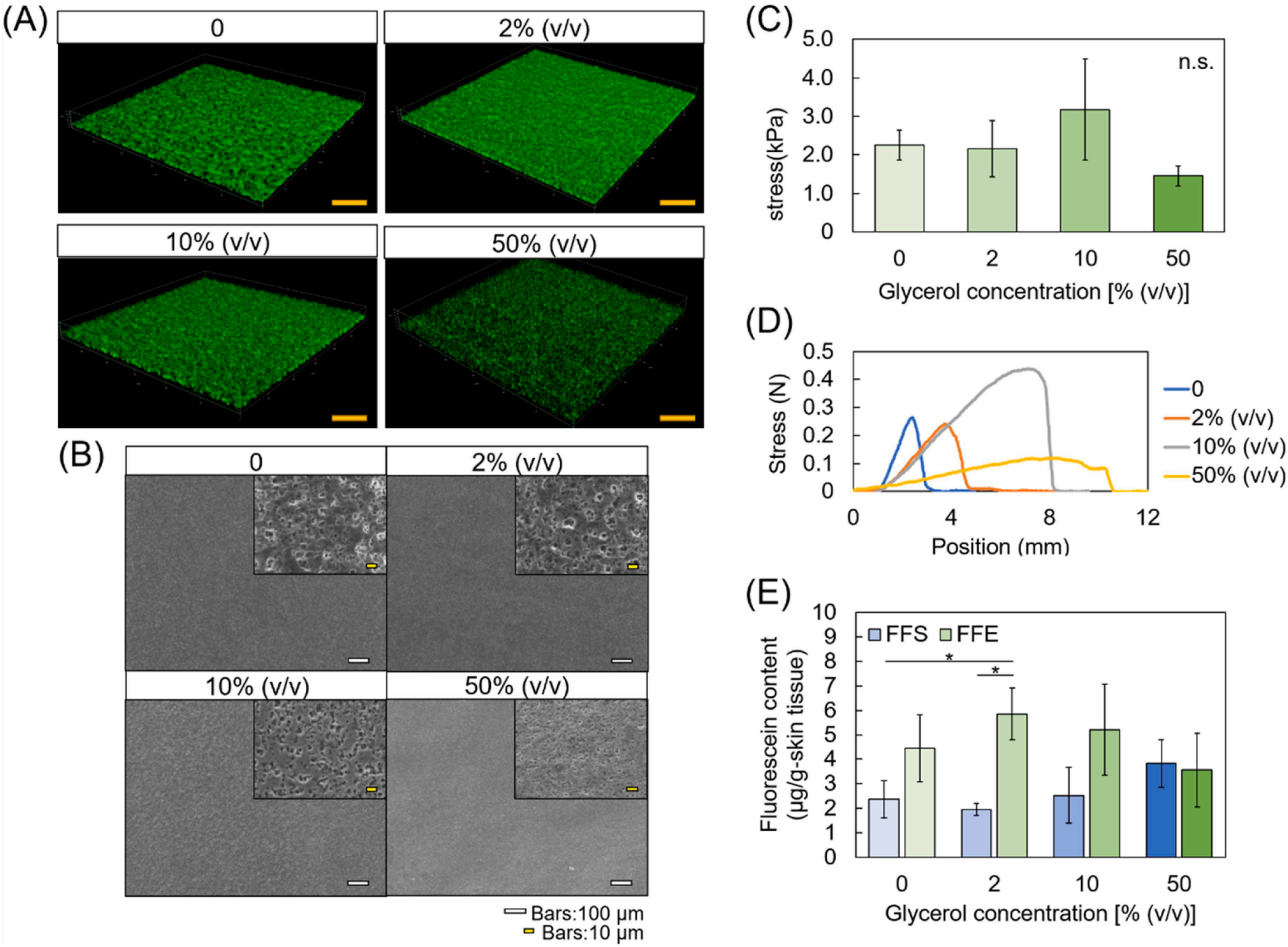
Optimisation of glycerol concentration in FFE. (A) CLSM images of FFE film. Bars: 50 µm. (B) SEM images of FFE film on the skin contact surface, Bars: 100 µm (white), Bars: 10 µm (yellow). (C) Skin adhesive of FFE at different glycerol concentrations, *n* = 3, n.s.=not significant. (D) Stress-strain curve of FFE at different concentrations. (E) Skin penetration of encapsulated FL from FFS/FFE at different glycerol concentrations, *n* = 3, *p < 0.05. CLSM: confocal laser scanning microscopy; SEM: scanning electron microscopy; FFE: film-forming emulsion; FL: fluorescein sodium; FFS: film-forming system.

After selecting 10% (w/v) PVA, we subsequently investigated the effects of glycerol content on stability, structure, skin adhesion, and *in vitro* skin penetration of encapsulated FL from FFE. Specifically, glycerol concentrations of 0, 2, 10, and 50% (v/v) were tested. No obvious differences in macroscopic creaming or phase separation were observed among the four formulations after storage at 4 °C and 25 °C for up to 144 h (Fig. S4). In Fig. 3A, green fluorescence indicates the distribution of the encapsulated W_G_/O-NE within the FFE film. No obvious differences in W_G_/O-NE droplet distribution were observed among the 0, 2, and 10% glycerol groups, and emulsion pathway-like structures were observed in all three groups. In contrast, in the 50% glycerol group, W_G_/O-NE droplets remained more uniformly dispersed, and interconnected pathway-like structures were less evident. The SEM images of the skin-contacting surfaces shown in Fig. 3B support these CLSM findings. The pore sizes in the 0, 2, and 10% groups were similar, whereas significantly smaller pores were detected in the 50% group.

As shown in Fig. 3C and D, the peel strengths of the films in the 0, 2, and 10% groups were comparable, whereas that of the 50% group exhibited a significant decrease. The release profiles from FFS and FFE films containing different glycerol concentrations are shown in Fig. 3E. In the FFS films composed of PVA and glycerol, no obvious difference in FL release was observed among the 0, 2, and 10% glycerol groups, whereas the 50% glycerol group showed a higher release. In the FFE films containing W_G_/O-NE, FL release from the 0, 2, and 10% glycerol groups was higher than that from the corresponding FFS films. In contrast, under the 50% glycerol condition, the difference in FL release between FFE and FFS was less evident.

### 3.4 *In vivo* penetration study

**Fig. 4:**
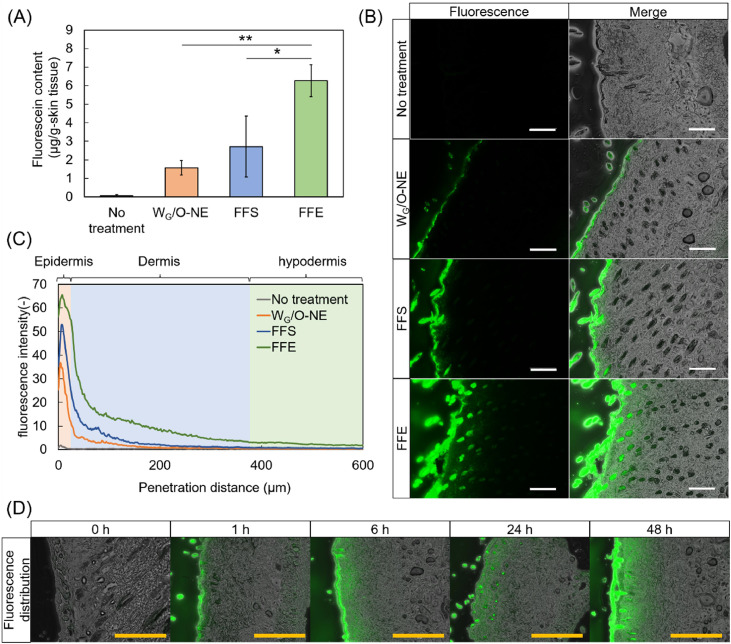
*In vivo* skin penetration of W_G_/O-NE, FFS, and FFE. (A) Quantitative fluorescence content in skin tissue normalized by skin weight, *n* = 3, *p < 0.05, **p < 0.01. (B) Visualization of fluorescence penetration into the skin, Bars: 200 µm. (C) Quantitative fluorescence intensity profiles as a function of penetration distance from the skin surface. (D) Visualization of the time-dependent skin penetration of fluorescein after application of fluorescein-loaded FFE for 0, 1, 6, 24, and 48 h, Bars: 400 μm. PVA: polyvinyl alcohol; FFE: film-forming emulsion; FFS: film-forming system; W_G_/O-NE: water-in-oil nanoemulsion.

Fig. 4A shows the amount of fluorescent model drug retained in the skin after different treatments, which was quantitatively evaluated as fluorescence content normalised by skin weight. Among the groups, FFE-treated skin exhibited the highest fluorescence retention, with 2.31-fold and 3.99-fold higher fluorescence levels than the FFS and W_G_/O-NE groups, respectively. These results indicate that the FFE formulation achieved the greatest retention of the fluorescent model drug in the skin. To further evaluate the skin penetration behavior, fluorescence images of mouse skin cross-sections are shown in Fig. 4B, and the corresponding fluorescence intensity profiles as a function of penetration distance from the skin surface are shown in Fig. 4C. Fluorescence in the W_G_/O-NE and FFS groups was mainly localised in the epidermis and dermis. In contrast, the FFE group showed stronger fluorescence intensity over a wider depth range, and fluorescence signals extended into the deeper skin region, including the subcutaneous tissue (more than 375 µm).

Time-dependent FL penetration after FFE application is shown in Fig. 4D, and the corresponding FL content retained in the skin is quantified in Fig. S5. At 1 h, the fluorescence signal was mainly observed near the epidermal region, and fluorescence was already detectable in the skin. After 6 h, green fluorescence was observed not only near the skin surface but also within the dermal region. For the 48 h group, the FFE film was replaced once at 24 h. Fluorescence signals remained visible in the skin at 24 and 48 h, and the quantitative results showed that FL remained detectable for up to 48 h under the repeated-application regimen.

### 3.5 Biocompatibility study

**Fig. 5:**
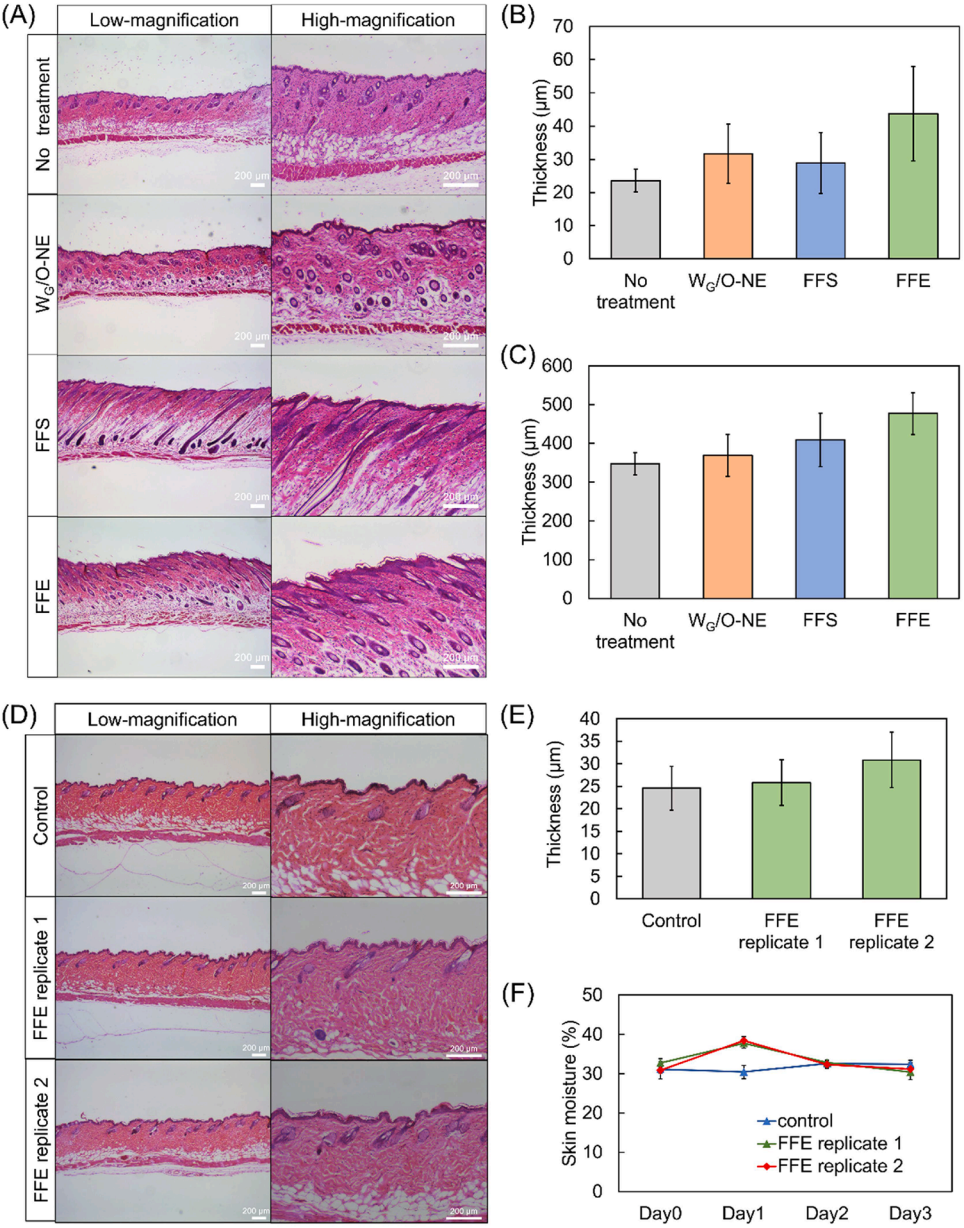
*In vivo* skin biocompatibility and recovery after treatment with W_G_/O-NE, FFS, FFE. (A) Histological evaluation with H&E staining, Bars: 200 µm. (B) Epidermal thickness, *n* = 3. (C) Dermal thickness, *n* = 3. (D) Skin recovery and safety of FFE after 2 d, Bars: 200 µm. (E) Epidermal thickness after 2 days. (F) Skin moisture level (FFE was applied on day 0 and removed on day 1). FFE: film-forming emulsion; FFS: film-forming system; W_G_/O-NE: water-in-oil nanoemulsion.

The biocompatibility of the W_G_/O-NE, FFS, and FFE formulations was assessed by histological and quantitative analyses. As shown in Fig. 5A, H&E staining revealed no visible pathological alterations or structural damage to either the epidermis or dermis in all treatment groups. Quantitative measurements in Fig. 5B and C show a mild increase in both epidermal and dermal thickness in all treatment groups compared to the untreated control, with the most pronounced thickening observed in the FFE group. However, by day 2 post-removal, epidermal thickness had returned to the baseline (Fig. 5D and E). Additionally, the skin moisture content increased after 24 h of FFE application but returned to normal levels within the subsequent 24 h (Fig. 5F).

## 4 Discussion

### 4.1 Preparation and characterisation of W_G_/O-NE and FFE

The particle size of the prepared W_G_/O-NE was consistent with that reported in our previous study [8]. A particle size below 300 nm and a PDI below 0.2 indicate that the prepared W_G_/O-NE had a homogeneous size distribution, which is suitable for use as a nanocarrier for skin delivery [20,21]. In addition, the EE of FL in W_G_/O-NE was 94.6 ± 0.4%, indicating that most of the model hydrophilic drug was retained in W_G_/O-NE before incorporation into FFE. Therefore, W_G_/O-NE was considered suitable as the drug-loaded internal phase for constructing the FFE system. However, the EE of FL in the final FFE could not be reliably determined because separating free FL from FL-loaded W_G_/O-NE within the viscous PVA/glycerol matrix may disrupt the nanoemulsion or induce FL redistribution during separation. This represents a methodological limitation of the present study. After incorporation into FFE, the formulation formed a transparent and flexible film on the skin surface. This film-forming behaviour can be attributed to the film-forming ability of PVA and the plasticising effect of glycerol [22,23]. The short-term macroscopic physical stability of FFE was temperature-dependent, with less creaming observed during refrigerated storage. Although no obvious instability was observed in the final formulation at 4 °C for 144 h, this observation period is insufficient to establish long-term storage stability. Comprehensive long-term stability studies under controlled storage conditions will therefore be required in future work.

Nanocarrier release from polymeric matrices is generally governed by the matrix network structure and particle–matrix interactions [24,25]. The recovery of W_G_/O-NE with a nanoscale particle size distribution after release from FFE suggests that the nanoemulsion could leave the film matrix while partially retaining its dispersed state. Moreover, the comparable FL release profiles of W_G_/O-NE and FFE indicate that incorporation into FFE did not create a substantial additional barrier to FL release. Together with the interconnected pathway-like structures observed by CLSM and SEM, these findings are consistent with the hypothesis that the proposed emulsion pathways facilitate the release of W_G_/O-NE and encapsulated FL from the FFE matrix. However, the present evidence does not directly demonstrate the three-dimensional continuity of these structures or confirm the transport of W_G_/O-NE through them. Further three-dimensional and dynamic structural analyses are therefore required to validate the proposed release mechanism.

Nevertheless, because FL is a small hydrophilic model molecule, the present penetration and retention results cannot be directly generalised to macromolecular drugs such as proteins or growth factors. Although previous studies demonstrated the delivery of growth factors using W_G_/O-NE, incorporation into the FFE matrix and subsequent film drying may affect the encapsulation, release, structural stability, and biological activity of macromolecular drugs. The recovery of W_G_/O-NE with a nanoscale particle size distribution after release from FFE provides a rationale for further investigation but does not directly demonstrate macromolecular delivery. Future studies should therefore evaluate hydrophilic macromolecules, including model proteins and growth factors, with respect to their encapsulation efficiency, structural and biological stability, release behaviour, and skin distribution.

### 4.2 Optimisation of PVA concentration in FFE

PVA concentration was a key formulation factor regulating the physical stability, internal microstructure, and functional performance of FFE. For the FFE system containing W_G_/O-NE droplets, the PVA matrix not only acted as a film-forming material but also contributed to restricting the migration and rearrangement of emulsion droplets during storage and drying. At lower PVA concentrations, especially 2.5% and 5%, the FFE solution exhibited relatively high fluidity. Under these conditions, micrometre-sized W_G_/O-NE droplets are prone to migration and aggregation because of insufficient spatial restriction by the polymer matrix and their interfacial instability [26]. This phenomenon may explain the more evident macroscopic creaming observed during storage at lower PVA concentrations (Fig. S3), as well as the larger and less uniform pores and interconnected structures observed inside the dried film and at the skin-contacting surface (Fig. 2A and B).

In contrast, increasing the PVA concentration enhances intermolecular entanglement and hydrogen bonding within the polymer matrix, thereby increasing matrix viscosity [27,28]. The reduced fluidity restricts droplet movement during storage and drying, which improves the macroscopic physical stability of FFE and suppresses excessive droplet aggregation. Consequently, smaller and more uniform porous microstructures were formed in the dried FFE films (Fig. 2A and B). This more uniform pore distribution at the skin-contacting surface may promote closer and more consistent contact between the FFE film and the skin. In addition, the increase in adhesion strength with increasing PVA concentration can be attributed to hydrogen bonding interactions between hydroxyl groups on PVA chains and biopolymers present in the skin [29]. Therefore, the enhanced skin adhesion observed at higher PVA concentrations may result from both the intrinsic adhesive properties of PVA and the formation of a more uniform skin-contacting interface.

However, increasing the PVA concentration was not necessarily favourable for drug release and skin penetration. The release of drugs or nanocarriers from hydrogel and polymeric matrices is known to be affected by the matrix network structure, diffusion pathways, and pore structure [23,29]. In the present study, drug release decreased at higher PVA concentrations (Fig. 2C), possibly because the denser and less connected microstructure increased the diffusion resistance of the drug within the FFE film. Therefore, optimisation of the PVA concentration in FFE should not simply aim to maximise either physical stability or skin penetration, but rather to balance droplet stability, film microstructure, skin adhesion, and drug delivery performance. Based on these considerations, a formulation containing 10% (w/v) PVA was selected for subsequent experiments because it provided a favourable balance among physical stability, skin adhesion, and skin penetration.

### 4.3 Optimisation of glycerol concentration in FFE

Glycerol concentration mainly affected the dried film structure, skin adhesion, and release behavior of FFE, whereas its influence on the short-term macroscopic physical stability of the liquid formulation was limited. The visual stability results showed no obvious differences in creaming or phase separation among the four glycerol concentrations (Fig. S4), suggesting that glycerol had a limited effect on the short-term macroscopic physical stability of FFE within this range.

In the dried FFE films, the 0, 2, and 10% glycerol groups showed similar W_G_/O-NE droplet distributions and pore structures, indicating that glycerol addition up to 10% did not markedly affect the formation of emulsion pathway-like structures. In contrast, the 50% glycerol group showed more uniform W_G_/O-NE droplet dispersion and smaller pores, with less evident interconnected pathways. This may be related to changes in the rheological properties of the FFE solution at high glycerol concentration, which could restrict local droplet aggregation during film drying and thereby reduce pathway formation.

The release results further support the effect of glycerol on the film structure. In the FFS films, the 50% glycerol group showed higher FL release, probably because excess glycerol increased the mobility of the polymer network and facilitated drug diffusion [30]. In the FFE films, FL release from the 0, 2, and 10% glycerol groups was higher than that from the corresponding FFS films, which may be attributed to the formation of emulsion pathways that provided additional diffusion routes through the W_G_/O-NE-derived microstructures and the interstitial spaces of the PVA network. In contrast, under the 50% glycerol condition, the difference between FFE and FFS became less evident, possibly because the formation of interconnected emulsion pathways was suppressed and the release behaviour was dominated by diffusion through the PVA/glycerol matrix.

The decrease in adhesion strength observed in the 50% glycerol group can be explained by the behaviour of glycerol as a small-molecule plasticiser. Glycerol can insert between PVA chains and increase the free volume and chain spacing within the polymer network [31,32]. However, when the glycerol concentration exceeds the compatibility threshold of the film, phase separation may occur, and excess glycerol can leach out from the film matrix [33,34]. Such leaching may lead to glycerol accumulation at the interface between the film and skin, ultimately reducing the adhesive strength of the FFE film in the 50% glycerol group. Therefore, although glycerol at an appropriate concentration improved film flexibility without disrupting emulsion pathway formation, excessive glycerol reduced skin adhesion and weakened the contribution of W_G_/O-NE to release enhancement. Based on these considerations, 10% (v/v) glycerol was selected as the optimal condition.

### 4.4 *In vivo* penetration study

The enhanced *in vivo* skin penetration of FFE can be attributed to the combination of prolonged skin residence, the penetration-enhancing function of W_G_/O-NE, and release from the film matrix. Film-forming systems are generally designed to form an adherent polymeric film on the skin after solvent evaporation, thereby prolonging formulation residence and supporting sustained dermal drug delivery [35]. In the present study, FFS increased skin fluorescence compared with W_G_/O-NE, probably because the film-forming matrix prolonged contact with the skin surface. However, fluorescence signals in the FFS group were mainly confined to the superficial skin layers, indicating that film formation alone was insufficient to achieve deep penetration.

In contrast, FFE combined the skin-retentive property of the film-forming matrix with the penetration-enhancing function of W_G_/O-NE. IPM-containing nanoemulsion systems can contribute to skin penetration enhancement [36], and the emulsion pathways formed within the FFE film may further allow W_G_/O-NE and the encapsulated fluorescent dye to be released from the film matrix. This combined effect likely contributed to the higher fluorescence retention and deeper fluorescence distribution observed in the FFE group.

Under the repeated-application regimen, the time-dependent penetration results support continued skin delivery. The progression of fluorescence from the epidermal region towards the dermal region, together with its detectability in the skin at later time points, is consistent with prolonged contact of FFE with the skin surface and continued release of W_G_/O-NE and FL from the film matrix.

### 4.5 Biocompatibility study

These results suggest that the observed stratum corneum thickening was transient and reversible within 2 days. After recovery, a loose basket-weave-like stratum corneum structure was observed on the skin surface, which is commonly regarded as a normal histological appearance of the stratum corneum [37]. Skin moisture content, a non-invasive parameter related to stratum corneum hydration and skin barrier condition [38], also returned to a level comparable to that of untreated skin within 24 h after FFE removal. The transient thickening may be associated with the presence of IPM, a known penetration enhancer that increases fluidity [39] or disrupts [40] the lipid structure of the stratum corneum to facilitate drug permeation. These mechanisms can also induce mild, reversible irritation. This can also be attributed to the occlusive nature of the FFE film, which limits transepidermal water loss and creates a hydrated microenvironment on the skin surface. Such hydration induces temporary swelling of corneocytes and partial separation of the intercellular lipid lamellae within the stratum corneum [41,42], contributing to apparent thickening.

Although epidermal thickness and skin moisture content recovered within the present observation period, skin status was not evaluated beyond 2 days after FFE removal. Therefore, delayed or longer-term irritation cannot be excluded, and future studies should include extended histological and skin barrier assessments at 7 days or later after film removal.

## 5 Conclusion

In this study, a novel FFE was developed by incorporating W_G_/O-NE into a PVA/glycerol film-forming matrix. Optimisation of the PVA and glycerol concentrations provided a practical balance among film integrity, skin adhesion, and permeability. Compared with W_G_/O-NE and FFS, the optimised FFE enhanced the skin retention and deeper distribution of the fluorescent model drug. It also exhibited time-dependent FL release and supported continued skin delivery under the tested repeated-application regimen. These findings demonstrate that FFE integrates the skin-retention properties of an *in situ*-formed film with the release and penetration-enhancing functions of W_G_/O-NE, providing a simple and adaptable platform for dermal and transdermal delivery.

The current formulation is intended primarily for intact skin because its ethanol content may limit its application to damaged or wounded skin. Moreover, the hydrophilic PVA-based film may lose adhesion or structural integrity under perspiration, high humidity, or water exposure. Future studies should develop ethanol-free formulations where appropriate, optimise the outer film-forming matrix to meet different application requirements, and evaluate therapeutically relevant cargos, including anticancer drugs, mRNA, and growth factors, in terms of their stability, release, skin delivery, safety, and therapeutic efficacy.

## CRediT authorship contribution statement

**Jia Hu:** Writing – review & editing, Writing – original draft, Methodology, Formal analysis, Data curation, Conceptualization. **Jannatul Fardous:** Writing – original draft, Writing – review & editing. **Yi Zhang:** Investigation. **Yasuhiro Ikegami:** Writing – review & editing, Investigation. **Hiroyuki Ijima:** Writing – review & editing, Supervision, Project administration.

## Declaration of competing interest

The authors declare that they have no known competing financial interests or personal relationships that could have appeared to influence the work reported in this paper.

## Data Availability

Data will be made available on request.

## Appendix Supplementary materials

Image, application 1
