# Supplementary material for: Design and characterisation of a film-forming emulsion combining enhanced skin permeability with prolonged drug delivery
Source: Biomater Biosyst. 2026 Aug 3;23:100144. doi: 10.1016/j.bbiosy.2026.100144 (PMC13485613; doi:10.1016/j.bbiosy.2026.100144)
Supplement: Supplementary file 1 [file mmc1.docx]

**Supplementary Material: Expanded Methods and Materials**

Jia Hu^a^, Jannatul Fardous^b^, Yi Zhang^a1^, Yasuhiro Ikegami^a^, Hiroyuki Ijima^a^*

^a^Department of Chemical Engineering, Faculty of Engineering, Graduate School, Kyushu University, 744 Motooka, Nishi-ku, Fukuoka 819-0395, Japan

^b^Department of Pharmacy, Faculty of Science, Comilla University, Comilla 3506, Bangladesh

^1^Institute for Materials Chemistry and Engineering, Kyushu University, 744 Motooka, Nishi-ku, Fukuoka 819-0395, Japan

∗Corresponding author:

Hiroyuki Ijima Email: [ijima@chem-eng.kyushu-u.ac.jp](mailto:ijima@chem-eng.kyushu-u.ac.jp)

Tel: +81-92-802-2748

Fax: +81-92-802-2748

Authors mailing address:

Jia Hu: [hu.jia.511@s.kyushu-u.ac.jp](mailto:hu.jia.511@s.kyushu-u.ac.jp)

Jannatul Fardous: [fardousj11@cou.ac.bd](mailto:fardousj11@cou.ac.bd)

Yi Zhang: [zhang.yi.651@m.kyushu-u.ac.jp](mailto:zhang.yi.651@m.kyushu-u.ac.jp)

Yasuhiro Ikegami: [yikegami@chem-eng.kyushu-u.ac.jp](mailto:yikegami@chem-eng.kyushu-u.ac.jp)

**Supplementary Note S1. Effect of ethanol on the stability of a simplified W/O/W nanoemulsion model**

**S1.1. Methods**

To evaluate whether ethanol affected the W/O/W emulsion structure formed during FFE preparation, a simplified PVA-free W/O/W nanoemulsion model was prepared using the same W_G_/O-NE as that incorporated into FFE. W_G_/O-NE was mixed with 50 mg/mL HCO-60 solution at a volume ratio of 1:5 and sonicated for 3 min. Absolute ethanol was subsequently added to achieve final ethanol concentrations of 0, 30, and 60% (v/v). The samples were stored at 4°C for 10 days. Before measurement, each sample was diluted 600-fold with ultrapure water. The mean hydrodynamic particle diameter and PDI were measured using the same DLS instrument and instrumental settings as described in Section 2.5.1 of the main manuscript.

**S1.2. Results**

As shown in Fig. S2, the mean particle diameter of the W/O/W nanoemulsion model remained approximately 150–180 nm in the presence of 0, 30, and 60% (v/v) ethanol throughout the 10-day observation period. The PDI remained below 0.25 in all groups, with no progressive increase in particle diameter or PDI indicative of marked aggregation.

**S1.3. Discussion**

These findings suggest that ethanol concentrations of up to 60% (v/v) did not markedly destabilise the W_G_/O-NE containing dispersion under the tested conditions. Because the final ethanol concentration in the prepared FFE was approximately 20% (v/v), the results provide supporting evidence that ethanol addition did not cause marked disruption of the dispersed W_G_/O-NE during FFE preparation. Nevertheless, this experiment used a simplified PVA-free dispersion and therefore does not reproduce all interactions within the complete FFE formulation.

**Supplementary Figures & Tables**

|  | | Composition | Concentration | Volume used (μL) |
| --- | --- | --- | --- | --- |
| Inner aqueous phase | Gelatin solution | Gelatin | 5% (w/v) | 900 |
|  | EDC/NHS solution | MES buffer | 0.05 M | 100 |
|  |  | EDC | 10 mg/mL |  |
|  |  | NHS | 6 mg/mL |  |
| Oil phase | | IPM | - | 2000 |
|  |  | ER-290 | 50 mg/mL |  |

Table S1: Composition and volumes used for W_G_/O-NE preparation.

|  | Composition | Concentration | Volume used (μL) |
| --- | --- | --- | --- |
| Inner phase | W_G_/O-NE | - | 500 |
| Film-forming phase | HCO-60 solution | 50 mg/mL | 500 |
|  | Absolute ethanol | - | 1000 |
|  | PVA solution | 16% (w/v) | 3000 |
|  | Glycerol | 10% (v/v, relative to PVA solution) | 300 |

Table S2: Composition and volumes used for FFE preparation. The final PVA concentration in the film-forming phase was 10% (w/v).

| Optimization study | PVA concentration  optimization | | | | | | Glycerol concentration  optimization | | | |
| --- | --- | --- | --- | --- | --- | --- | --- | --- | --- | --- |
| Formulation | P1 | P2 | P3 | P4 | P5 | P6 | G1 | G2 | G3 | G4 |
| PVA concentration [% (w/v)] | 2.5 | 5 | 7.5 | 10 | 12.5 | 15 | 10 | 10 | 10 | 10 |
| Glycerol concentration [% (v/v)] | 10 | 10 | 10 | 10 | 10 | 10 | 0 | 2 | 10 | 50 |

Table S3: FFE formulations used for PVA and glycerol optimization.

Figure S1: Schematic illustration of the proposed transdermal drug delivery mechanism of the film-forming emulsion (FFE). W_G_/O-NE: water-in-oil nanoemulsion.

Figure S2. Effect of ethanol concentration on the stability of a simplified PVA-free W/O/W nanoemulsion model. Bars represent particle diameter, and lines represent PDI. Data are presented as the mean ± SD, n = 3. W/O/W: water-in-oil-in-water. W_G_/O-NE: water-in-oil nanoemulsion.

Figure S3: Representative photographs of FFE formulations with different PVA concentrations during short-term storage at 25 °C and 4 °C. P1–P6 correspond to FFE formulations containing 2.5, 5, 7.5, 10, 12.5, and 15% (w/v) PVA, respectively. All formulations contained 10% (v/v) glycerol. Samples were stored undisturbed and photographed at 0.5, 1, 2, 4, 24, 48, 72, and 144 h for visual assessment of macroscopic physical stability, including phase separation, creaming, sedimentation, and precipitation. PVA: polyvinyl alcohol; FFE: film-forming emulsion.

Figure S4: Representative photographs of FFE formulations with different glycerol concentrations during short-term storage at 25 °C and 4 °C. G1–G4 correspond to FFE formulations containing 0, 2, 10, and 50% (v/v) glycerol, respectively. All formulations contained 10% (w/v) PVA. Samples were stored undisturbed and photographed at 72 and 144 h for visual assessment of macroscopic physical stability, including phase separation, creaming, sedimentation, and precipitation. PVA: polyvinyl alcohol; FFE: film-forming emulsion.

Figure S5: Time-dependent FL content retained in skin after FFE application. FL content was quantified at the indicated time points and normalised to skin weight (μg/g skin; n = 3). The 1, 6, and 24 h groups received a single application, whereas the 48 h group received a second FFE application at 24 h.
